# Improving access to alcohol use disorder pharmacotherapy and treatment in primary care settings

**DOI:** 10.1186/1748-5908-10-S1-A75

**Published:** 2015-08-20

**Authors:** Hildi Hagedorn, Alex Sox-Harris, Jennifer Wisdom, Donald Hugh Myrick, Michael Dawes, Randall Brown, Elizabeth Oliva

**Affiliations:** 1 Substance Use Disorders Quality Enhancement Research Initiative, Minneapolis Veterans Affairs Health Care System, Minneapolis, MN 55417, USA; 2 Department of Psychiatry, University of Minnesota School of Medicine, Minneapolis, MN 55455, USA; 3 Substance Use Disorders Quality Enhancement Research Initiative, Palo Alto Veterans Affairs Health Care System, Menlo Park, CA 94025, USA; 4 Department of Health Policy, George Washington University, Washington, D.C. 20052, USA; 5 Center for Drug and Alcohol Problems, Ralph H. Johnson Veterans Affairs Medical Center, Charleston, SC 29401, USA; 6 Department of Psychiatry and Behavioral Services, Medical University of South Carolina, Charleston, SC 29425, USA; 7 Substance Abuse Treatment Program, South Texas Veterans Affairs Health Care System, San Antonio, TX 78229, USA; 8 Department of Psychiatry, University of Texas Health Science Center at San Antonio, San Antonio, TX 78229, USA; 9 William S. Middleton Memorial Veterans Hospital, Madison, WI 53705, USA; 10 Department of Family Medicine, University of Wisconsin School of Medicine and Public Health, Madison, WI 53715, USA

## 

Despite the high prevalence of alcohol use disorders (AUDs), in a given year, only 12.1% of those meeting diagnostic criteria receive any treatment. Most individuals with AUDs are identified in primary care settings and referred to substance use disorders clinics, however only a minority attend treatment services. Developing options for treatment within primary care settings may increase receipt of services for AUDs. Safe and effective pharmacological treatments exist that could be integrated into primary care settings. This study will refine, implement and evaluate an intervention to integrate AUD treatment options, particularly pharmacological options, into primary care settings in three large Veterans Health Administration facilities.

The current paper will present the implementation and evaluation strategies and the results of the developmental portion of the formative evaluation (FE). The implementation intervention targets multiple stakeholders: 1) substance use disorder and primary care mental health integration providers trained as local implementation leaders, 2) primary care providers who will have access to consultation, educational materials, a dashboard of patients with AUD on their caseload, and feedback on their prescribing practices, and 3) Veterans diagnosed with AUD who will receive educational mailings. Evaluation methods will combine FE with an interrupted time series to monitor change in facility level prescribing rate. The developmental FE consists of interviews with the local implementation leaders, primary care providers, and Veterans with AUD diagnoses. The Consolidated Framework for Implementation Research (CFIR) informed the development of the interview guides. Qualitative analysis will identify CFIR constructs that function as significant barriers and facilitators to implementation success and results will be used to refine the intervention plan. The findings will advance implementation science by demonstrating the use of theory to inform refinement of an implementation intervention and will contribute to accumulating knowledge regarding the relevance of specific CFIR constructs to implementation success.

